# Acute amyloid-β exposure disrupts insulin signaling in hCMEC/D3 blood–brain barrier endothelial cells

**DOI:** 10.1186/s12987-026-00825-2

**Published:** 2026-06-19

**Authors:** Douglas A. Nelson, Suresh K. Swaminathan, Vaishnavi Veerareddy, Hannah S. Seo, Samira M. Azarin, Krishna R. Kalari, Karunya K. Kandimalla

**Affiliations:** 1 Department of Pharmaceutics and Brain Barriers Research Center, College of Pharmacy, University of Minnesota, Minneapolis, MN 55455 USA; 2 Department of Chemical Engineering and Materials Science, College of Science and Engineering, University of Minnesota, Minneapolis, MN USA; 3 Department of Quantitative Health Sciences, Mayo Clinic, Rochester, MN USA

**Keywords:** Blood–brain barrier, Alzheimer’s disease, Insulin signaling, Amyloid-β, Endothelial cells, Akt signaling, Reverse-phase protein array

## Abstract

**Background:**

Brain insulin resistance and cerebrovascular dysfunction emerge early in late-onset Alzheimer’s disease, but how amyloid-β (Aβ) disrupts insulin signaling at the cerebrovascular blood–brain barrier—a major site of insulin receptor signaling and transport into the brain—remains unclear.

**Methods:**

We exposed two distinct human blood-brain-barrier endothelial cell models to soluble Aβ40 or Aβ42 for 1 h, followed by 100 nM insulin for 10 min. Protein and phosphoprotein responses were quantified by reverse-phase protein array, and differential expression was evaluated using linear models.

**Results:**

In hCMEC/D3 cells, Aβ40 reduced insulin-stimulated Akt activation and converted insulin’s normal inhibition of AMPK into modest stimulation. Aβ42 did not alter insulin-stimulated Akt signaling but moderately suppressed basal Akt activation. Because the iBMEC model did not show insulin-induced activation of PI3K–Akt signaling, mechanistic interpretation of Aβ effects on insulin-responsive signaling focused on hCMEC/D3 cells.

**Conclusions:**

These findings identify acute Aβ40-sensitive impairment of insulin-responsive Akt signaling in hCMEC/D3 BBB endothelial cells. The results define candidate signaling nodes for further mechanistic study of Aβ effects on BBB insulin-signaling dysfunction.

**Supplementary Information:**

The online version contains supplementary material available at https://doi.org/10.1186/s12987-026-00825-2.

## Background

Alzheimer’s disease (AD) is a multifaceted neurodegenerative disorder characterized by the accumulation of amyloid-β (Aβ) peptides and neurofibrillary tangles of hyperphosphorylated tau [1–4]. In late-onset AD, these Aβ and tau pathologies consistently co-occur with cerebrovascular dysfunction and brain insulin resistance, both emerging early in the disease process [5–7].

Among the cerebrovascular changes associated with late-onset AD, dysfunction of the blood–brain barrier (BBB) is especially important. The BBB forms the primary interface between blood and brain, regulating nutrient delivery and clearance of metabolic waste, including toxic Aβ peptides [8]. The BBB is an integral component of the neurovascular unit (NVU), an interdependent association of neurons, astrocytes, pericytes, and cerebral endothelium that couples neuronal activity to local changes in cerebral blood flow [9]. In AD, the BBB and broader NVU exhibit early and progressive dysfunction [10].

Metabolic syndrome and type 2 diabetes are major risk factors for AD [11]. Chronic peripheral insulin resistance disrupts metabolic insulin signaling, leading to impaired energy metabolism and elevated inflammation in the brain that accelerates AD progression [12]. Notably, insulin receptors are enriched in brain microvascular endothelial cells compared to other NVU components, positioning the BBB endothelium as the primary site for insulin entry and signaling within the NVU [13]. Impaired insulin signaling at the BBB is thought to disrupt neurovascular coupling and significantly reduce Aβ clearance from the brain [9, 12, 14]. These findings suggest a positive feedback loop: Aβ impairs endothelial insulin signaling, which further limits Aβ clearance [12, 14]. Understanding how Aβ exposure and insulin resistance drive BBB dysfunction at the molecular level is therefore critical.

The two most abundant Aβ isoforms, Aβ40 and Aβ42, are characterized by distinct pathological distributions: Aβ40 predominates in vascular amyloid deposits associated with cerebral amyloid angiopathy, while Aβ42, although less abundant overall, is the dominant component of parenchymal plaques [15, 16]. The two isoforms also exert distinct effects on the cerebrovascular endothelium, but the mechanisms and isoform specificity of Aβ interference with insulin signaling remain poorly defined [14, 17].

Prior studies show that Aβ can impair insulin signaling in both neurons and BBB endothelial cells, but the mechanistic basis for this disruption—and whether Aβ40 and Aβ42 differ in their effects on BBB insulin transduction—remains unknown [14, 17]. To characterize the direct effects of Aβ exposure, we examined the acute response to Aβ40 and Aβ42 on BBB insulin signaling. Identifying these transient molecular changes is essential because acute pathway perturbations often reveal the mechanistic entry points that lead to degenerative processes, even though the chronic phenotypes may differ markedly [18–21]. Accordingly, these early signaling changes can provide insight into how chronic endothelial dysfunction and brain insulin resistance can emerge. To capture these acute responses within a broader signaling context, we utilized reverse-phase protein arrays (RPPA), which enable parallel measurement of hundreds of proteins and phosphoproteins. This study, therefore, defines how acute exposure to Aβ40 and Aβ42 perturbs BBB endothelial insulin signaling, establishing the mechanistic context needed to evaluate isoform-specific contributions to vascular insulin resistance.

## Methods

### Aβ and insulin peptides

Aβ40 and Aβ42 were obtained from Aapptec (Louisville, KY). Soluble Aβ40 and Aβ42 solutions were prepared according to the procedure described by Klein and characterized as described in our earlier publications [22]. Briefly, Aβ films were formed by dissolving the lyophilized peptide in HFIP and evaporating the solvent under vacuum. The resultant films were hydrated by sequential addition of 50 µL DMSO, 50 µL deionized water, and 100 µL pH 8.3 F12 medium, with brief sonication after each step. The solutions were centrifuged at 10,000 rpm for 5 min at 4 °C to remove any insoluble aggregates, and the supernatant was diluted in cell culture medium to final a concentration of 1 µM, as assayed by ELISA. Vehicle controls were prepared using the same DMSO, water, and medium sequence without Aβ peptide. The final DMSO concentration in vehicle- and Aβ-containing treatment medium was 0.25% v/v.

Insulin powder (Sigma, Saint Louis, MO) was solubilized in 0.1 N sodium carbonate (5.3 mg/mL). A 1000 µM master stock was generated at 5.808 mg/mL and stored at 4 °C. From this, a final concentration of 100 nM was prepared in the cell culture medium, and the cells were treated with 100 nM insulin for 10 min.

### Cell cultures

Human cerebral microvascular endothelial cell (hCMEC/D3) line was a generous gift from Pierre-Olivier Couraud of the Institut Cochin (Paris, France). The endothelial cells were cultured according to the protocols provided by the Couraud group [23]. Polarized hCMEC/D3 cell monolayer were cultured by seeding the cells at passage 30 on 24 mm Transwells as detailed in our earlier publications [24].

Brain-specific microvascular endothelial-like cells (iBMECs) were differentiated from IMR90-4 (WiCell, Madison, WI) human induced pluripotent stem cells (hiPSC), according to the procedures reported by Stebbins et al. [25]. On day 8, iBMECs were transferred to collagen IV/fibronectin (CN IV-FN)–coated 24.6 mm CellTreat^®^ inserts (CELLTREAT Scientific Products, Pepperell, MA) at a concentration of 1 × 106 cells/cm2. The iBMECs were cultured in endothelial medium consisting of Human Endothelial Serum-Free Medium (Thermo Fisher Scientific) supplemented with 1% platelet-poor plasma derived serum (PDS; Biomedical Technologies), 20 ng/mL basic fibroblast growth factor (PeproTech), and 10 µM retinoic acid (Millipore Sigma). On day 9, cells were maintained in Human Endothelial Serum-Free Medium containing only 1% PDS.

For coculturing, frozen human brain vascular pericytes (hBVPs; ScienCell, Carlsbad, CA) were thawed and expanded in poly-D-lysine (PDL)-coated T-75 flasks using Dulbecco’s Modified Eagle’s Medium (DMEM, Millipore Sigma) supplemented with 10% fetal bovine serum (FBS; Thermo Fisher Scientific). Human astrocytes (HA; ScienCell, catalog #1800) were expanded per vendor instructions in human astrocyte growth medium (Cell Applications, Inc.) on PDL-coated plates. Pericytes and astrocytes were then seeded at a 1:1 ratio by cell number onto the bottoms of PDL-coated 6-well plates at a total cell density of 312,500 cells per well in iBMEC medium. After iBMECs formed a barrier, the inserts were placed into the wells containing pericytes and astrocytes to establish a non-contact BBB model; cultures were maintained at 37 °C in a humidified 5% CO_2_ atmosphere. Pericyte and astrocyte co-cultures were employed to support BBB-like endothelial phenotype during model establishment; downstream RPPA signaling analyses were restricted to endothelial cell lysates.

### Treatments with Aβ and insulin

Treatments and lysate preparation for iBMECs and hCMEC/D3 cells were the same. At the end of each treatment, the spent medium was removed, and cells were washed twice with DPBS. Then DMEM (2.5 mL) was added to the abluminal side. One milliliter of either control medium or Aβ (1 µM) was added luminally and incubated for 1 h. Where indicated, cells were spiked with 100 nM insulin for the last 10 min before the termination of the experiment. All Aβ and insulin incubations were carried out on the luminal side.

Treatment groups included untreated medium, vehicle, insulin (10 min), Aβ40 (1 h), Aβ42 (1 h), Aβ40 (1 h) plus insulin, and Aβ42 (1 h) plus insulin. Each of the seven conditions had three replicates, totaling 21 wells across seven treatments. Plates 1 (untreated) and 2 (insulin) were processed in tandem; followed by Plates 3 (Aβ40 alone) and 4 (Aβ40 plus insulin); Plates 5 (Aβ42 alone) and 6 (Aβ42 plus insulin); and plate 7 (vehicle only). These groupings defined four independent RPPA runs (batches).

At the end of treatment, plates were placed on ice. Cells were immediately washed once with ice-cold HBSS, followed by the addition of ice-cold HBSS containing protease and phosphatase inhibitors. Then RIPA lysis buffer containing protease and phosphatase inhibitors (50 µL per well) was added. Cells were scraped with a cell scraper and collected in pre-chilled microcentrifuge tubes. This sequence was repeated for all wells. Lysates were sonicated for 4 min until clear and centrifuged at 10,000 rpm for 10 min at 4 °C. The supernatants were transferred to fresh 0.5 mL tubes, pellets discarded, and samples stored at − 20 °C. For final preparation, 4X SDS without bromophenol blue and with beta-mercaptoethanol was added, samples were boiled for 5 min, and stored at − 80 °C.

### Reverse phase protein array (RPPA) assays

Lysates for RPPA were collected from endothelial cells on the Transwell inserts. RPPA was performed by the MD Anderson Functional Proteomics RPPA Core (Set151; 21 samples; 306 antibodies). Lysates were blotted as five two-fold dilutions (undiluted, 1:2, 1:4, 1:8, 1:16) on nitrocellulose slides. Then the blots were probed using tyramide amplification, developed with DAB, scanned on a Huron TissueScope, and quantified in Array-Pro. Relative protein levels for each antibody were estimated by SuperCurve interpolation of the five-point dilution series. The Core provided sample-specific loading correction factors CF1 and CF2, where CF1 is calculated within the submitted set and CF2 across all samples printed on the slide; samples with CF2 < 0.25 or > 2.5 are flagged as protein-loading outliers recommended for exclusion. The hCMEC/D3 samples had CF1 0.83–1.35 and CF2 0.45–0.72, whereas iBMEC samples had CF1 0.66–1.33 and CF2 0.56–1.10. None of the samples met CF2 outlier criterion and were not excluded.

### RPPA data analysis

Sample-wise median centering was applied after inspection of raw expression distributions and variance profiles across samples. Probes corresponding to proteins that are not biologically relevant to the BBB endothelium were excluded. The exclusion list was curated from established literature and cross-referenced for lack of functional abundance based on median values from pseudobulk analysis of a public transcriptomic dataset (NCBI GEO: GSE163577). A list of filtered probes is provided in Supplementary List 1. A total of 268 probes were included after filtering, out of which 62 were phospho-probes.

Principal component analysis (PCA) was performed for each two-group contrast to assess replicate clustering before differential expression analysis. Cluster coherence was quantified using silhouette widths computed on the principal components explaining ≥ 95% of the variance, and Mahalanobis distances in two dimensions using a pooled within-class covariance estimate.

To reduce the influence of high-variance and outlier probes, differential expression was fit with Limma using empirical-Bayes moderation configured with a mean–variance trend (trend=TRUE) to accommodate RPPA’s intensity-dependent variance and robust hyperparameter estimation (robust=TRUE). We estimated sample precision weights with arrayWeights for each affected contrast. Benjamini-Hochberg multiple hypothesis correction was applied per contrast.

To generate composite scores that emphasize probes supported by both meaningful effect size and statistical evidence, we transformed differential expression statistics into bounded scores using raised-cosine functions with log₂-fold change (LFC) parameters log2(1.1) and log2(1.5), and FDR parameters −log10(0.10) and −log10(0.001). Composite scores were then calculated as the geometric mean, penalizing imbalance between the two components.

To evaluate inter-batch shifts, we defined a set of invariant total-protein probes based on within-batch comparisons. A probe was considered invariant if, within each of the three two-group batches (untreated vs. insulin, Aβ40 vs. Aβ40 + insulin, Aβ42 vs. Aβ42 + insulin), the estimated log₂ fold-change between groups is ± 0.1 within a 95% confidence interval. Confidence intervals were calculated from the Limma model using empirical Bayes-moderated standard errors. Only probes meeting this criterion in all three batches were retained. Probes with any plausible mechanism for acute Aβ sensitivity were excluded from the invariant set. For each inter-batch comparison, we summarized the distribution of differences across the invariant set by reporting the Wilcoxon signed-rank p-value, the median log₂ fold-change, and the median absolute deviation (MAD).

### Western blot and silver stain

Silver staining of Aβ samples was performed using the Pierce Silver Stain Kit (Thermo Fisher Scientific). Samples (10 µl) were mixed with Laemmli sample buffer (4.5 µl) and β-mercaptoethanol (0.5 µl), then resolved on 4–15% Mini-PROTEAN^®^ TGX™ precast gels (Bio-Rad, #456–1086) at 200 V for 30 min. Gels were processed according to the manufacturer’s protocol. SeeBlue™ Plus2 prestained protein standard (Invitrogen™, #LC5925) was used as a molecular weight marker. The resulting gel is shown in Supplementary Fig. 1.

For Western blot analysis, lysates prepared as described above were quantified by BCA assay, and equal amounts of protein (25–30 µg) were resolved by SDS–PAGE and transferred to membranes as described previously [26]. Membranes were probed with antibodies against phospho-Akt (Ser473) (1:1000, Cell Signaling Technology) and total Akt (1:1000, Cell Signaling Technology). Band intensities were quantified using Image Studio Lite (v5.2), and phospho-Akt signals were normalized to total Akt.

## Results

### Differential expression overview and quality control

We used reverse-phase protein arrays (RPPA) with 268 antibody probes to quantify protein and phosphoprotein abundance in two BBB endothelial models. Cells were exposed to vehicle, Aβ40, or Aβ42 (1 µM, 1 h), with or without insulin (100 nM, 10 min), with three biological replicates per condition across four RPPA batches. We then established quality control metrics, examined Aβ effects in the absence of insulin, validated insulin responses in both models, and assessed how Aβ modulates insulin-stimulated signaling.

We assessed replicate quality and treatment separation using principal component analysis. hCMEC/D3 samples exposed to Aβ plus insulin showed reduced within-group coherence and increased variability (Supplementary Table 1), so we applied sample-specific precision weights in differential expression analysis for these contrasts. Table 1 summarizes the number and direction of significantly changed probes (FDR < 0.05) across eight key comparisons, capturing the scale and directionality of responses across basal Aβ contrasts, insulin-only responses, Aβ-dependent insulin-response shifts, and final insulin-stimulated state comparisons. Aβ40 consistently produced differential expression in more proteins than Aβ42 in corresponding contrasts across iBMECs and in non-insulin comparisons for hCMEC/D3. The hCMEC/D3 vehicle − untreated comparison showed substantially elevated differential expression (103 probes).

**Table 1:** Counts of differentially expressed probes (FDR < 0.05) by direction in hCMEC/D3 (HD3) and iBMEC models across eight key contrasts. Upregulated (up) indicates positive log₂ fold-change; downregulated (dn) indicates negative log₂ fold-change. “Insulin shift” refers to second-order contrasts quantifying how Aβ modulates the insulin response: (Aβ with insulin − Aβ) − (insulin − untreated)

Contrast	HD3up	HD3dn	HD3tot	iBMECup	iBMECdn	iBMECtot
Vehicle − untreated	27	76	103	2	7	9
Aβ40 − vehicle	59	5	64	38	27	65
Aβ42 − vehicle	34	9	43	7	9	16
insulin − untreated	30	52	82	14	28	42
Aβ40 insulin shift	7	10	17	45	25	70
Aβ40ins − insulin	5	13	18	20	12	32
Aβ42 insulin shift	21	17	38	5	0	5
Aβ42ins − insulin	9	20	29	5	2	7

**Fig. 1:**
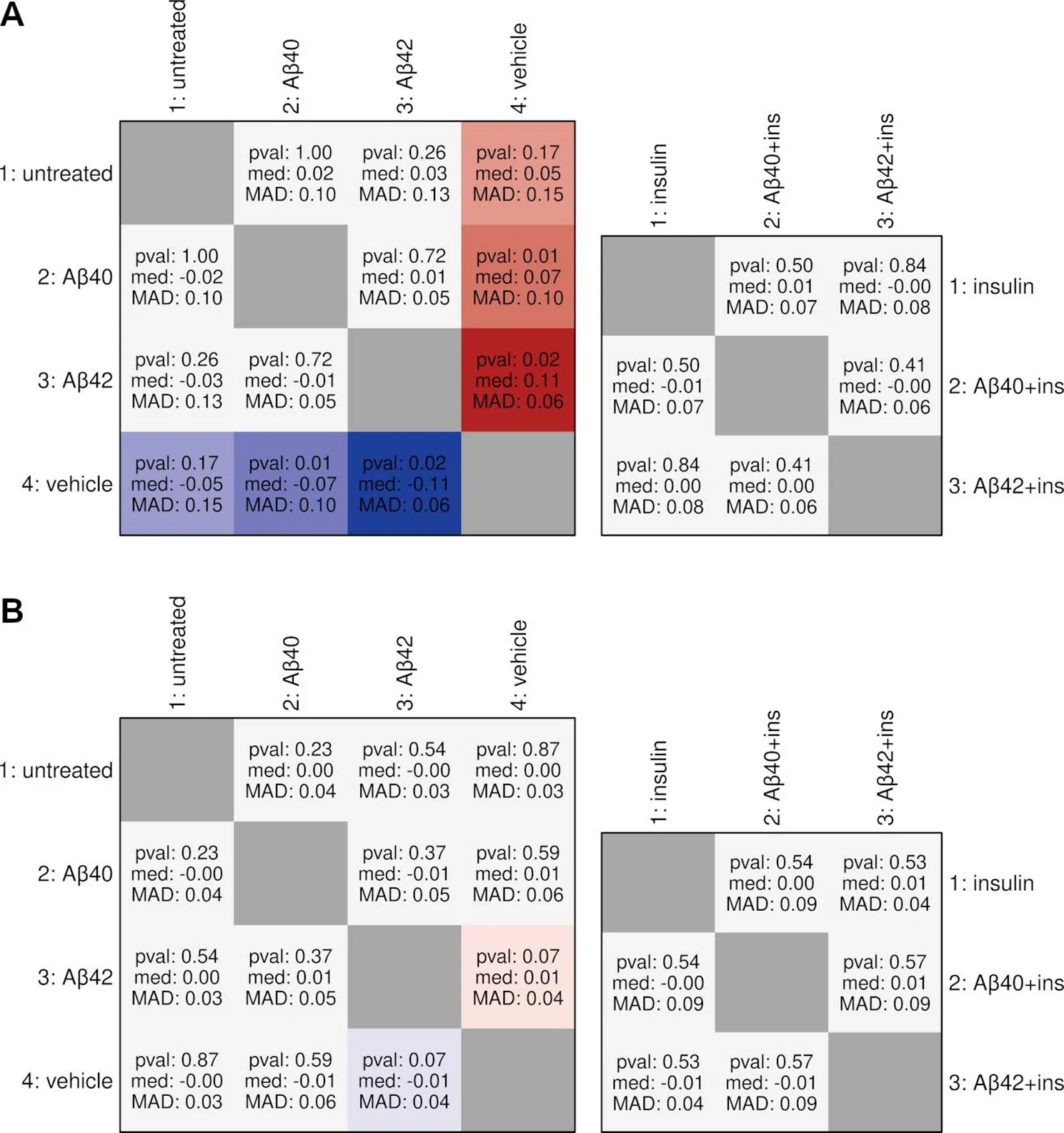
Evaluation of inter-batch shifts using invariant total-protein probes. RPPA analyses were run in four batches: (1) untreated and insulin, (2) Aβ40 and Aβ40 with insulin, (3) Aβ42 and Aβ42 with insulin, and (4) vehicle alone. Invariant probes were defined as total-protein probes with 95% confidence intervals for log_2_ fold-change within ± 0.1 in all three two-group batches. This intra-batch invariant set was used to assess batch-to-batch consistency. **A** (top) shows hCMEC/D3 results: the left matrix is a 4 × 4 comparison of all pairwise inter-batch shifts among the non-insulin groups (untreated, Aβ40, Aβ42, vehicle), and the right matrix is a 3 × 3 comparison among the insulin-containing groups (insulin, Aβ40 with insulin, Aβ42 with insulin). **B** (bottom) shows the corresponding iBMEC results with the identical structure. Each cell reports three statistics (top-to-bottom): Wilcoxon signed-rank p-value (pval), median log₂ fold-change (med), and median absolute deviation (MAD). Cells are colored when pval < 0.20 and |med| ≥ 0.01. Red indicates positive shifts and blue indicates negative shifts, defined as the row group minus the column group; color intensity scales with the magnitude of the median shift. Diagonal cells represent comparisons of a batch to itself and are shaded gray

To assess residual batch effects after normalization, we identified invariant total-protein probes showing stable abundance within batches and examined their consistency across batches (Fig. 1). Twelve invariant probes were identified in hCMEC/D3 and 53 in iBMECs. Inter-batch comparisons showed general stability in both models, except for the hCMEC/D3 vehicle batch, which exhibited systematic negative displacement. This provides a batch-level explanation for the elevated number of differentially expressed probes in the hCMEC/D3 vehicle − untreated contrast Table 1.

**Fig. 2:**
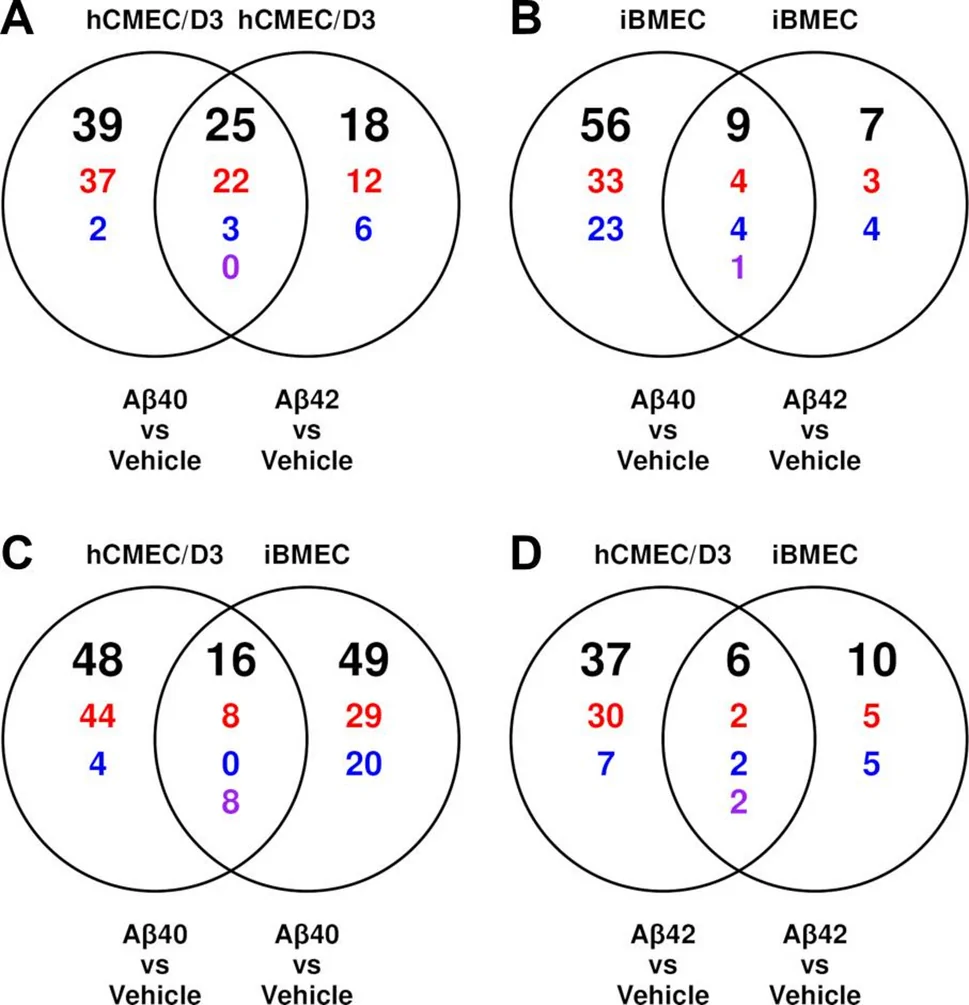
Overlap of differentially expressed proteins between Aβ isoforms and between BBB endothelial models. Differential expression is defined by FDR < 0.05, with direction based on log₂ fold-change sign. Black numbers above each diagram indicate total differentially expressed proteins. Colored numbers denote directional overlap: red for up/up, blue for down/down, and purple for opposite-sign overlaps. Top: Aβ isoform overlaps for Aβ-vehicle contrasts in hCMEC/D3 (**A**) and iBMECs (**B**). Bottom: cell model overlaps for Aβ-vehicle contrasts with Aβ40 (**C**) and Aβ42 (**D**)

Venn overlap analysis of differentially expressed probes (Fig. 2) showed that, within each model, the two Aβ isoforms exhibited modest overlap: at least half of Aβ42-responsive probes were also differentially expressed in the same direction under Aβ40. Cross-model concordance was lower, with only one-eighth (Aβ40) and one-quarter (Aβ42) of iBMEC responses replicated in hCMEC/D3.

### Aβ effects without insulin

**Fig. 3:**
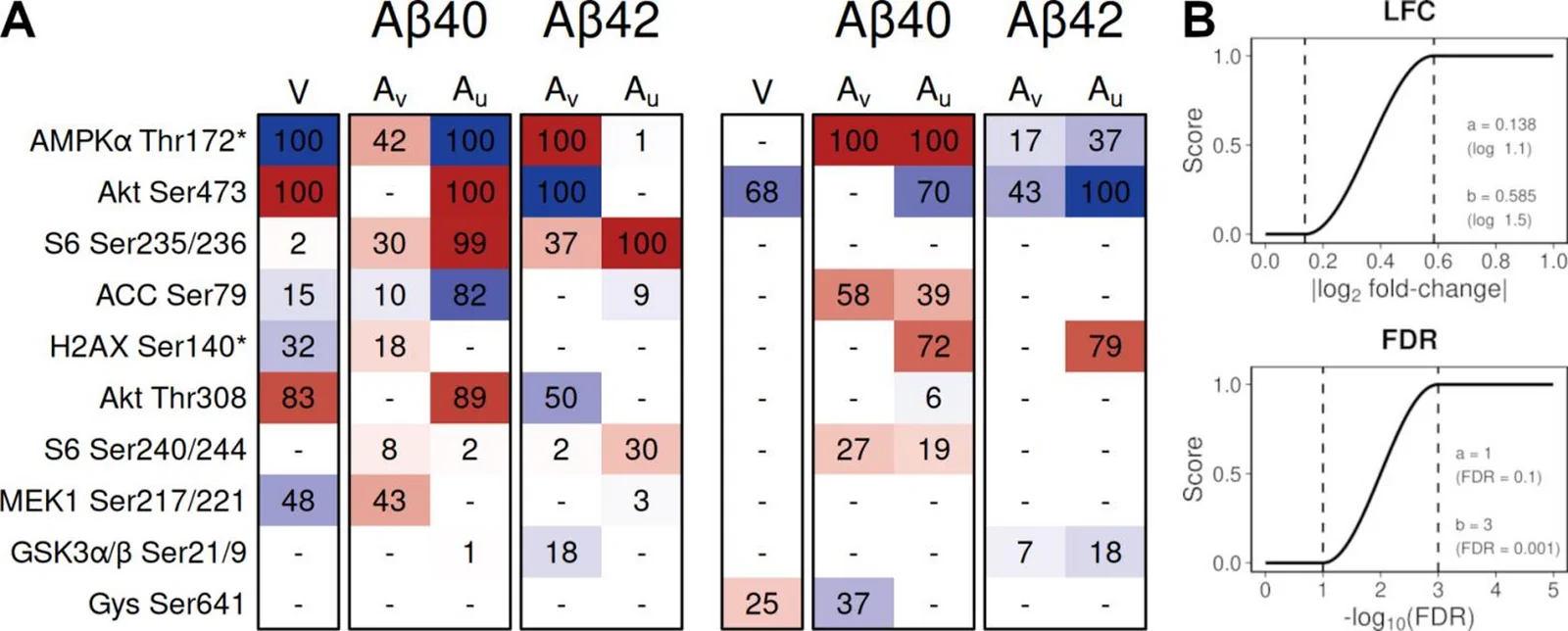
Differentially expressed phosphoprotein probes in BBB endothelial models after one-hour Aβ exposure. **A**: Heatmap matrices show differentially expressed phosphoproteins in hCMEC/D3 (left) and iBMEC (right). Each model shows the vehicle − untreated contrast (V) and, for each Aβ isoform, Aβ − vehicle (Av) and Aβ − Untreated (Au). Each cell contains a composite score computed as the geometric mean of two raised cosine functions—one based on |log_2_ fold-change| (LFC) and one on −log_10_(FDR)—each scaled to [0, 1], with final scores multiplied by 100. Red indicates upregulation and blue indicates downregulation. Only the ten highest-scoring probes are shown. Cells marked with “–” indicate a composite score of 0, corresponding to |LFC| < 0.138 or FDR > 0.1. Antibody probes marked with an asterisk (*) were not validated by the MD Anderson RPPA Core. **B**: Raised cosine scoring functions. For LFC (top), the transition occurs between a = 0.138 (log₂ 1.1) and b = 0.585 (log₂ 1.5). For FDR (bottom), the transition occurs between a = 1 (FDR = 0.1) and b = 3 (FDR = 0.001)

Before examining how Aβ modulates insulin signaling, we characterized Aβ effects in the absence of insulin to establish baseline responses. To distinguish Aβ-specific from solvent effects, we analyzed three contrasts: vehicle − untreated (V), Aβ − vehicle (Av), and Aβ − untreated (Au), where “untreated” denotes cells in culture medium alone and “vehicle” denotes medium containing 0.25% DMSO used for Aβ preparation. Because Aβ treatments include the same vehicle, the Aβ − vehicle contrast isolates Aβ-specific effects. Figure 3 summarizes the top phosphoprotein changes in response to one-hour amyloid exposure in both BBB endothelial models. The score matrix integrates fold-change and FDR into a single metric reflecting magnitude and statistical reliability. Vehicle exposure upregulated Akt Ser473 and Akt Thr308 relative to untreated controls, opposite to the direction of vehicle batch bias. In the hCMEC/D3 model, Aβ42 reversed this upregulation. For other phosphoproteins, we focused on those showing consistent directional changes in both the Aβ − vehicle and Aβ − untreated contrasts. In hCMEC/D3 cells, both Aβ isoforms upregulated S6 Ser235/236 and S6 Ser240/244, while Aβ40 reduced ACC Ser79. In iBMECs, Aβ40 upregulated AMPKα Thr172, ACC Ser79, and S6 Ser240/244, whereas Aβ42 downregulated AMPKα Thr172, Akt Ser473, and GSK3α/β Ser21/9.

### Insulin signaling in hCMEC/D3 and iBMEC models

**Fig. 4:**
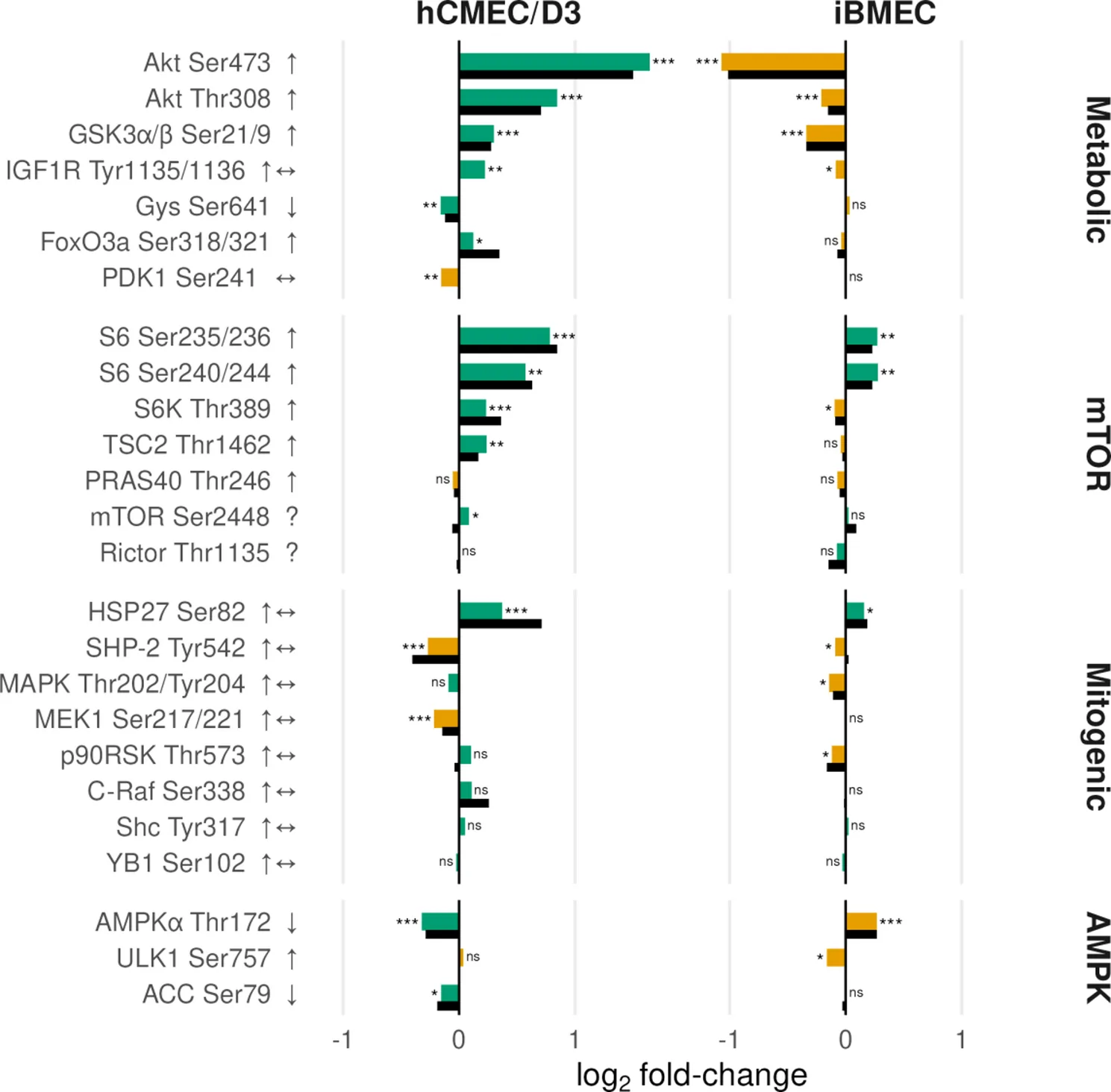
Log_2_-fold changes in insulin signaling probes in hCMEC/D3 and iBMEC BBB models. Cells were treated with 100 nM insulin for 10 min and compared with untreated controls. Probes are grouped into four functional pathways: metabolic, mTOR, mitogenic, and AMPK. Literature-based canonical direction acute insulin-response is shown to the right of each probe (↑ increase, ↓ decrease, ↔ stable, ? no expectation). Colored bars represent observed log₂ fold-change: green indicates consistency with expectation, yellow/orange indicates divergence. Asterisks denote statistical significance (*, **, *** for FDR < 0.05, < 0.01, < 0.001; ns = not significant). Black bars represent phospho-to-total protein ratios. Probes lacking black bars have no corresponding total-protein measurement (IGF1R Tyr1135/1136, ULK1 Ser757, Shc Tyr317, YB1 Ser102). For PDK1 Ser241, the phospho-to-total ratio is near unity (zero on log scale)

Proteins with differential phosphorylation after 10-minute exposure to 100 nM insulin in both BBB endothelial models are shown in Fig. 4. These phosphoproteins are organized into four functional pathway groups: metabolic (Akt and immediate substrates), mTOR-centered (S6/S6K/TSC2/PRAS40/mTOR/Rictor), mitogenic (MEK-ERK cascade), and AMPK. Results are shown as log₂ fold-changes for phosphoproteins, with phospho/total protein ratios displayed where corresponding total protein measurements were available. Phospho/total ratios were generally consistent with phosphoprotein changes alone, except for PDK1 Ser241, where the ratio remained near unity as expected given its constitutive phosphorylation. The hCMEC/D3 model exhibited canonical insulin responses across metabolic and mTORC1 pathways. Akt Thr308 and Akt Ser473 showed the strongest activation, with corresponding increases in downstream mTOR-axis substrates (S6 Ser235/236, S6K Thr389, and TSC2 Thr1462). AMPKα Thr172 and ACC Ser79 decreased, consistent with insulin-mediated AMPK suppression. In contrast, the iBMEC model showed non-canonical insulin responses, with key metabolic nodes such as Akt Thr308 and GSK3α/β Ser21/9 changing in the opposite direction to expected. Subsequent analyses of Aβ effects on insulin signaling therefore focus exclusively on the hCMEC/D3 model.

### Aβ effects on insulin signaling

**Fig. 5:**
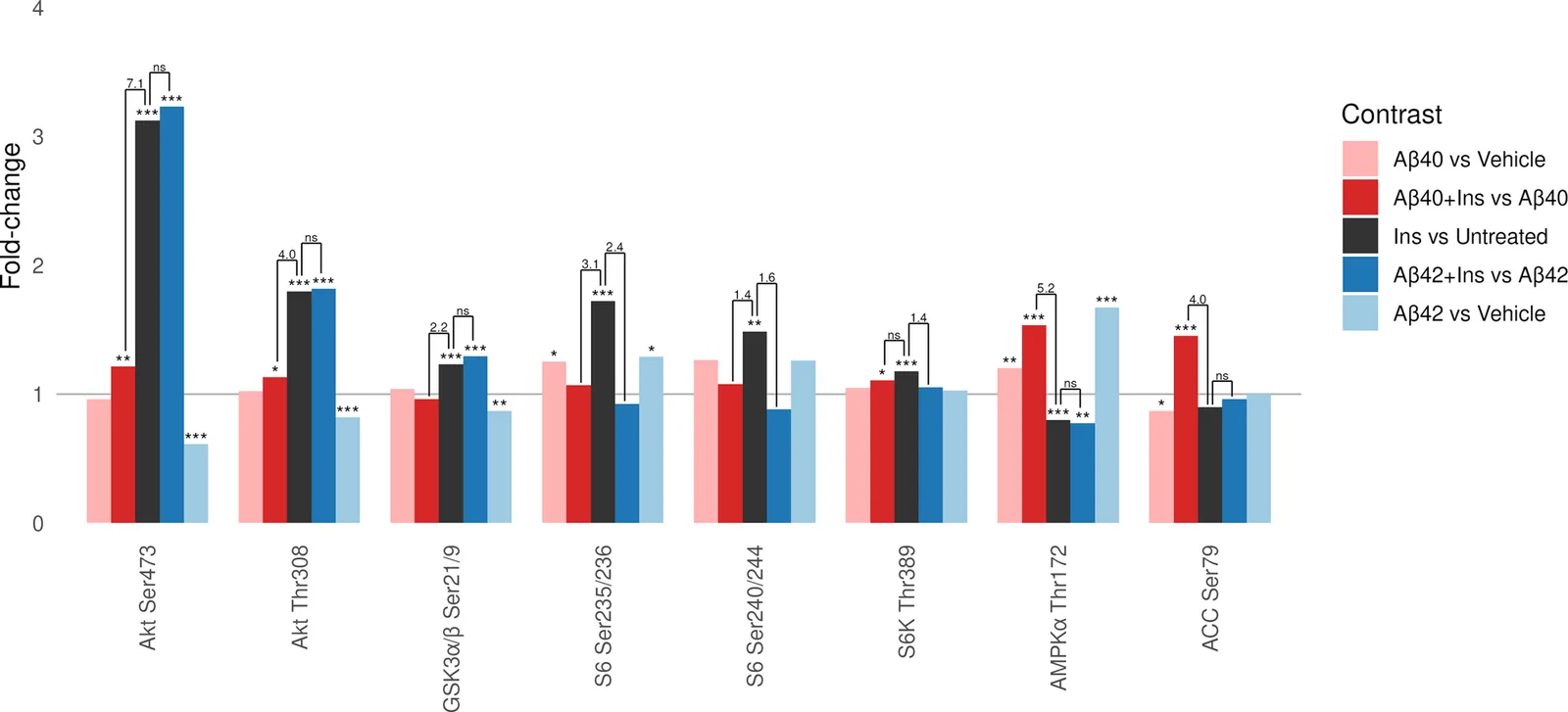
Insulin-responsive probes in hCMEC/D3 BBB endothelial cells with and without Aβ exposure. Shown are selected metabolic, mTOR, and AMPK probes with significant insulin responses. Insulin effects are shown in untreated cells and in cells exposed to Aβ40 or Aβ42 (both with DMSO vehicle). For reference, Aβ − vehicle contrasts without insulin are also shown. The horizontal line at fold-change = 1 denotes no change (log_2_ fold-change = 0) within each contrast. Asterisks (*, **, ***) denote FDR < 0.05, < 0.01, and < 0.001, respectively; ns = not significant. Numeric annotations above comparison brackets indicate −log_10_(FDR) from second-order contrasts, each bracket denoting the significance of differences between the effect of insulin on untreated cells and its effect on Aβ-treated cells

**Fig. 6:**
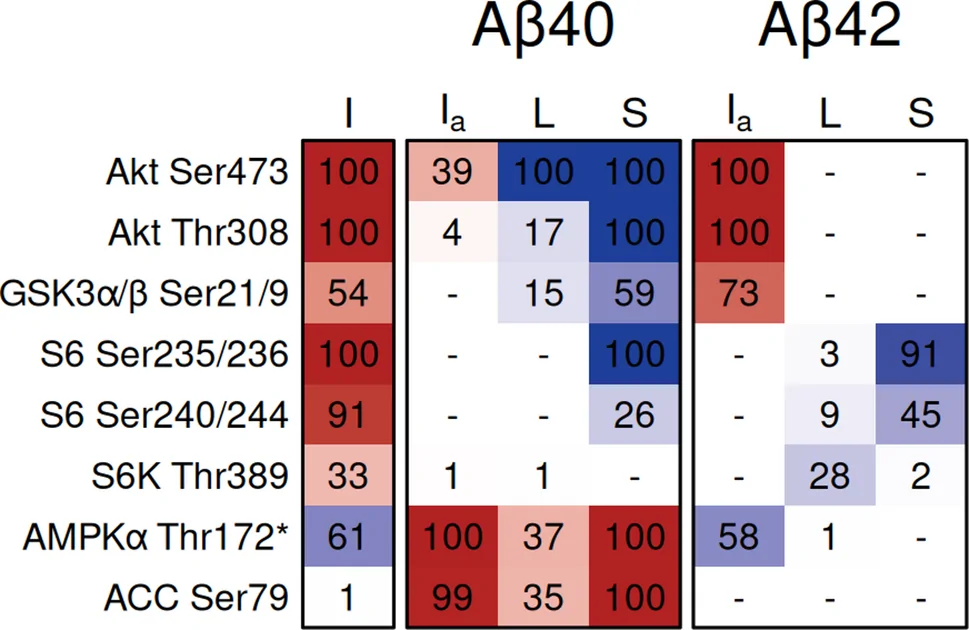
Differentially expressed insulin-responsive phosphoproteins in hCMEC/D3 with Aβ40 and Aβ42 exposure. Contrasts: I (insulin − untreated), I_a_ (insulin with Aβ − Aβ), L (level difference: insulin with Aβ − insulin), and S (second-order contrast measuring the shift in insulin response: I_a_ − I). Each cell contains a score computed as the geometric mean of two functions—one based on log₂ fold-change and one on FDR—each scaled to [0, 1]. Final scores are multiplied by 100. Red indicates upregulation and blue indicates downregulation. Antibody probes marked with an asterisk (*) were not validated by the MD Anderson RPPA Core

To quantify how Aβ modulates insulin signaling in hCMEC/D3 cells, we examined insulin responses in the presence and absence of Aβ. We used second-order contrasts [(Aβ with insulin − Aβ) − (insulin − untreated)] to measure whether Aβ amplifies, attenuates, or reverses insulin-stimulated changes at each phosphoprotein node. Figure 5 shows fold-changes for five contrasts involving Aβ and insulin effects, with statistical comparisons showing the second-order contrasts. To distinguish between attenuation, augmentation, reversal, and ambiguous outcomes, we integrated the second-order comparison with three additional insulin-related contrasts: I = insulin − untreated, Iₐ = Aβ plus insulin − Aβ, and L = Aβ plus insulin − insulin (Fig. 6). These four contrasts were interpreted using decision rules formalized in Supplementary Fig. 2. Table 2 summarizes the integrated interpretation for key insulin-responsive phosphoproteins.

Aβ40 produced the most substantial alterations in insulin signaling. Within the metabolic pathway, Aβ40 attenuated insulin-stimulated Akt activation at both Ser473 and Thr308, with corresponding attenuation of its downstream target GSK3α/β Ser21/9. This interpretation was supported by the integrated contrast framework shown in Fig. 6: in addition to the second-order insulin-response contrast, the level contrast L showed lower final Akt phosphorylation after Aβ40 plus insulin than after insulin alone for Akt Ser473 (FDR = 0.002) and Akt Thr308 (FDR = 0.01). In the AMPK pathway, Aβ40 reversed insulin’s suppression of AMPKα Thr172 and its direct substrate ACC Ser79, instead producing net stimulation; this interpretation was also supported by the level contrast L for AMPKα Thr172 (FDR = 0.0004) and ACC Ser79 (FDR = 0.008). Both Aβ40 and Aβ42 reduced insulin-stimulated phosphorylation of S6 Ser235/236 and S6 Ser240/244, with Aβ42 also reducing S6K Thr389.

**Table 2:** Summary effects on insulin signaling after one-hour treatment by Aβ40 or Aβ42 in hCMEC/D3 cells based on the four contrasts in Fig. 6. Interpretations are assigned by the decision rules in Supplementary Fig. 2. Robust attenuation for Akt Ser473 indicates fold change < 2/3 and 0.001 FDR < 0.001 (Fig. 3B) for both L and S contrasts

Probe	Aβ40	Aβ42
Akt Ser473	Robust attenuation of insulin stimulation.	No effect on insulin response.
Akt Thr308	Attenuation of insulin stimulation.	No effect on insulin response.
GSK3α/β Ser21/9	Attenuation of insulin stimulation.	No effect on insulin response.
S6 Ser235/236	Attenuation of insulin stimulation, or saturation following basal stimulation.	Attenuation of insulin stimulation.
S6 Ser240/244	Attenuation of insulin stimulation, or saturation following basal stimulation.	Attenuation of insulin stimulation.
S6K Thr389	No effect on insulin response.	Attenuation of insulin stimulation.
AMPKα Thr172	Reversal of insulin inhibition.	No effect on insulin response.
ACC Ser79	Reversal of insulin inhibition.	No effect on insulin response.

To independently validate the principal Akt finding, we performed Western blot analysis of Akt Ser473 phosphorylation following Aβ40 exposure and insulin stimulation. Consistent with the RPPA results, Aβ40 reduced the insulin-stimulated increase in Akt Ser473 phosphorylation relative to insulin-stimulated untreated cells. Quantification of pAkt Ser473 normalized to total Akt confirmed a significant insulin response in control cells and an attenuated response after Aβ40 exposure (Supplementary Fig. 3).

## Discussion

### Mechanistic routes to BBB insulin resistance: receptor loss, IRS inhibition, and direct Aβ–INSR interaction

BBB insulin resistance in late-onset AD has been linked to reduced cerebrovascular INSR availability, inhibitory serine phosphorylation of IRS proteins, and direct Aβ interaction with INSR. In human AD microvessels, defective cerebrovascular INSR signaling has been associated with reduced vascular INSRα-B, a higher INSRα-A/B ratio, and higher BACE1 burden [27]. In vitro work in porcine primary and mouse brain endothelial cell models showed that Aβ40 and Aβ42 reduce the INSR functional subunit (IR-β) and LRP1 levels by accelerating autophagy–lysosomal degradation, thereby impairing insulin-mediated signaling [14].

In the present study, total INSR protein levels were unchanged after 1 h Aβ exposure. The present findings and prior reports of receptor loss reflect observations at different time scales of Aβ–INSR interaction: the present study captures acute signaling responses in the presence of intact receptors, whereas prior work reflects longer-term consequences in which receptor depletion impairs insulin signaling.

Although total cellular INSR abundance was unchanged in whole-cell lysates, altered receptor localization or integrity could produce functionally similar outcomes to receptor depletion without affecting total protein abundance detectable by RPPA. We therefore consider two general insulin resistance mechanisms: (1) inhibitory serine phosphorylation of IRS proteins driven by kinase pathways engaged during Aβ exposure, and (2) direct Aβ–INSR interaction, which could alter receptor activation or signal output.

Aβ is known to induce oxidative stress, particularly through direct engagement with the RAGE receptor, which activates NADPH oxidase and other ROS-generating pathways [28]. Resulting oxidative stress can activate stress kinases (JNK1/2, IKKβ, PKCβII) that phosphorylate IRS at inhibitory serine sites [29, 30]. In parallel, MAPK-RSK and p70S6K (S6K) can phosphorylate IRS independently of oxidative stress [29, 31]. Evidence for IRS serine phosphorylation is inconclusive, however, as functional IRS probes were absent from the panel, and the measured phosphorylating kinases (JNK1, PKCβII, MAPK, S6K) showed no activation at 1 h, although activation at earlier time points cannot be excluded.

Experiments using human placental membranes and purified insulin receptor preparations showed that Aβ40 and Aβ42 reduce insulin binding and inhibit insulin-stimulated receptor autophosphorylation in a manner consistent with competitive inhibition [32]. Both isoforms engage the insulin-binding ectodomain with relatively low affinity, but within physiologically relevant concentrations [32]. Structural modeling of monomeric Aβ40 also supports an energetically favorable but weaker interaction at the receptor ectodomain [33]. Lower affinity binding, however, does not simply produce weaker responses. For example, insulin and IGF-family ligands can elicit qualitatively distinct signaling profiles despite engaging the same family of receptors [34]. Because INSR activation depends on coordinated conformational transitions and multisite autophosphorylation, Aβ interaction with the receptor could alter signaling branch balance or produce incomplete receptor activation rather than simply acting as a weak insulin mimetic, although the downstream consequences remain poorly characterized.

### Basal Aβ responses: separating biological patterns from vehicle-batch effects

Given RPPA’s semi-quantitative nature, our analytical approach—including invariant-probe batch assessment, dual-contrast validation, and composite scoring—prioritized identifying robust qualitative patterns for targeted validation while minimizing false positives. The invariant probe set analysis (Fig. 1) revealed systematic negative displacement in the hCMEC/D3 vehicle batch. This bias affects interpretation of vehicle-related contrasts: proteins appearing downregulated in vehicle − untreated or upregulated in Aβ − vehicle comparisons align with the observed direction of displacement in probes that are invariant within each batch and are therefore interpreted more cautiously. However, several patterns oppose this bias direction or are additionally supported by non-vehicle contrasts, and therefore are more consistent with biological effects than technical artifacts.

Aβ40 consistently produced stronger responses than Aβ42 across both models. This cannot be attributed to systemic batch shift, since the pattern appears in both cell models and both contrasts share the same vehicle reference (Figs. 2 and 3). This pattern aligns with Aβ40’s greater vasculotropic character [35]. Additionally, phosphorylation of Akt at S473 and T308 is robustly increased in the vehicle − untreated comparison, which is opposite in direction to the vehicle batch shift (Fig. 3) and consistent with reports of DMSO-induced Akt activation [36].

To identify robust patterns, we focused on phosphoproteins showing consistent directional changes in both Aβ − vehicle and Aβ − untreated comparisons (Fig. 3). In the hCMEC/D3 model, phosphorylation of S6 increased with both Aβ40 and Aβ42 in both contrasts, while in iBMECs, Aβ40 upregulated S6 Ser240/244 in both contrasts. Together these results suggest Aβ-induced mTORC1 activity, as S6 Ser240/244 is a canonical mTORC1 readout [37]. The absence of changes in S6K Thr389 or Akt phosphorylation at 1 h may reflect a kinetic mismatch, as phosphorylation at these sites is often more transient than at S6.

In hCMEC/D3 cells, Aβ40 also produced ACC Ser79 dephosphorylation in both contrasts. This suggests reduced AMPK activity as ACC is a direct AMPK substrate [38]. Reduced AMPK relieves mTORC1 inhibition, providing an additional mechanism consistent with increased S6 phosphorylation [39]. In contrast, iBMECs showed coordinated upregulation of both AMPKα Thr172 and ACC Ser79 with Aβ40.

Akt downregulation at Ser473 following Aβ42 treatment also opposes the batch-shift direction and appears in both iBMEC contrasts and the hCMEC/D3 Aβ42 − vehicle comparison, while that isoform showed no effect on insulin-stimulated Akt (Table 2). This basal-only inhibition suggests potential interference with β1-integrin–regulated PI3K/Akt signaling maintained by adhesion to basement-membrane proteins. This pathway represents a major constitutive source of basal Akt activity in adherent cells such as BBB endothelium, consistent with evidence that oligomeric Aβ42 directly binds and perturbs β1-integrin function [40–44].

### Insulin-only responses define model suitability: canonical hCMEC/D3 signaling and divergent iBMEC responses

Before assessing Aβ effects on insulin signaling, we examined insulin alone (100 nM, 10 min vs. untreated) to confirm expected pathway activation and establish which endothelial model exhibited a canonical insulin-responsive signaling profile under the experimental conditions used here. The two models were included because they represent complementary BBB endothelial systems: hCMEC/D3 is a simpler, extensively characterized human cerebral microvascular endothelial model with documented BBB-associated markers and insulin receptor expression, whereas the iBMEC-based coculture system provides a more complex, higher-resistance barrier configuration.

The insulin-responsive nodes measured span interconnected metabolic and mitogenic pathways (diagram in Supplementary Fig. 4). In the metabolic pathway, insulin binding to INSR activates IRS proteins, which serve as a critical regulatory node upstream of PI3K-Akt [45]. Akt is activated by phosphorylation at Thr308 and Ser473 and coordinates multiple downstream branches: it inhibits GSK3α/β at Ser21/9 to support glycogen synthesis and BBB maintenance [46, 47], suppresses the energy sensor AMPK through phosphorylation of AMPKα at Ser485/491 [48], and activates mTORC1, which in turn activates S6K and ribosomal protein S6 to stimulate protein synthesis [49, 50]. AMPK directly phosphorylates ACC at Ser79, suppressing lipid synthesis [48]. Insulin can also activate the mitogenic MEK-ERK-RSK cascade, through which RSK provides an Akt-independent route to mTORC1 activation and can directly phosphorylate S6 at Ser235/236 [37].

In hCMEC/D3 cells, probes associated with PI3K–Akt, AMPK, and mTOR signaling followed a canonical insulin response pattern, with Akt phosphorylated at Ser473 and Thr308 showing the largest increases, consistent with signal amplification through the PI3K–Akt axis [51]. Decreases in AMPKα Thr172 and ACC Ser79 reflect a metabolic shift toward anabolism, while increases in S6, p70S6K, and TSC2 phosphorylation are consistent with mTORC1 activation [52, 53]. IGF1R showed mild activation despite its low insulin affinity [54], consistent with the supraphysiological dose, while PDK1 Ser241 remained stable as expected for constitutive activation-loop phosphorylation [55]. Mitogenic pathway proteins (MAPK/ERK) showed minimal change, consistent with transient, context-dependent insulin-induced ERK activation in endothelial cells [56–58]. In contrast, the iBMEC model did not recapitulate a physiological insulin response under the conditions used here, with key metabolic nodes such as Akt Thr308 and GSK3α/β Ser21/9 changing in directions opposite to expectation. This early iBMEC model, which has since been refined [59], was therefore not considered suitable for mechanistic interpretation of Aβ effects on insulin-responsive signaling, and subsequent analyses focused on hCMEC/D3 cells. The cause of this divergence was not determined in the present study and may reflect differences in differentiation state, extracellular matrix, media composition, or co-culture configuration [59, 60].

### Aβ effects on insulin responsiveness: Aβ40 attenuation of Akt signaling and reversal of AMPK suppression

Aβ modulation of insulin signaling showed isoform-specific and pathway-specific patterns (Table 2). Aβ40 uniquely attenuated insulin-stimulated Akt and downstream GSK3α/β phosphorylation while reversing insulin’s suppression of AMPK and ACC. This effect is supported by both second-order insulin-response contrasts and level contrasts comparing final insulin-stimulated states, which show lower Akt Ser473 and Thr308 phosphorylation after Aβ40 plus insulin relative to insulin alone and preclude apparent attenuation arising from saturation of phosphorylation sites following the DMSO-mediated increase in basal activation. Both isoforms appeared to blunt S6 responses, although from an Aβ-induced elevated baseline.

Direct Aβ-INSR interactions have been shown to depend on aggregation state, with monomeric and oligomeric species exhibiting different receptor-binding behaviors and downstream signaling effects [33]. Aβ40 is relatively stable in monomeric form and, when it aggregates, proceeds more directly toward protofibrils and fibrils with relatively low oligomer accumulation, whereas Aβ42 rapidly forms small, stable oligomeric assemblies [61]. Although aggregation states were not comprehensively characterized, silver staining performed 1 h after preparation, matching the exposure duration used here, showed Aβ40 migrating predominantly at a molecular weight consistent with monomer (~ 4 kDa), whereas Aβ42 exhibited strong presence of higher-molecular weight species (Supplementary Fig. 1). Prior studies from Kandimalla and colleagues characterized Aβ40 and Aβ42 aggregation states using antibody-based assays and surface plasmon resonance, supporting the interpretation that Aβ40 preparations are predominantly monomeric whereas Aβ42 preparations are enriched in soluble oligomeric species [62–66]. Thus, aggregation-state differences produce distinct Aβ species with the potential to engage INSR differently, offering a plausible explanation for why only Aβ40 inhibited insulin-induced Akt activation.

Prior work from our group demonstrated that both Aβ40 and Aβ42 reduce insulin-stimulated Akt phosphorylation in hCMEC/D3 cells [67], whereas the present RPPA analysis identifies Aβ40, but not Aβ42, as attenuating insulin-responsive Akt phosphorylation. This discrepancy likely reflects differences in experimental context between the two studies.

A major methodological distinction is culture configuration. Earlier studies were conducted in plate-based monolayers on standard 6-well plastic surfaces, whereas the present study employed a Transwell BBB model with defined luminal and abluminal compartments. This configuration introduces endothelial polarity and compartmentalization, which can alter membrane accessibility, receptor distribution, lipid-domain organization, endocytic trafficking, and extracellular-matrix and junctional signaling—parameters that directly influence PI3K–Akt activity. These parameters are particularly relevant for soluble Aβ42, whose oligomeric forms, through multivalent interactions and increased avidity, are more sensitive than monomers to membrane microenvironment, including surface clustering, lipid-domain organization, integrin–FAK basal Akt tone, and endocytic uptake and routing.

In addition, prior studies incorporated overnight low-serum conditioning before Aβ and insulin exposure and defined the control condition as DMEM without DMSO solvent, whereas the present study did not include a serum starvation step and used a DMSO-containing vehicle control. These differences can shift basal PI3K–Akt activity and insulin responsiveness, thereby affecting the observed Akt response.

The Aβ42-specific pattern—inhibition of basal tone but not insulin stimulation—should nevertheless be interpreted cautiously, as they were not reproduced in prior studies using different models and experimental conditions, suggesting that the observed response is context dependent with yet unknown generalizability.

Both isoforms elevated basal S6 phosphorylation and blunted insulin-stimulated S6 responses. The similar final S6 phosphorylation levels observed with insulin following Aβ treatment and insulin alone suggest saturation of phosphorylation sites. S6K can also initiate negative feedback through serine phosphorylation of IRS, which could blunt later insulin activation, but only Aβ40 treatment shows the expected Akt inhibition.

Aβ40’s reversal of insulin-mediated AMPK suppression could involve not only loss of Akt-dependent inhibition, but also some source of AMPK activation. Under normal conditions, insulin restrains AMPK through Akt phosphorylation of AMPKα at Ser485/491, preventing Thr172 activation [48]. When Aβ40 attenuates insulin-stimulated Akt activation, this inhibitory restraint is relieved, allowing AMPK to remain active despite insulin exposure. This loss of inhibition could be coupled with stimulation through energy-sensing (LKB1) or Ca²⁺-dependent (CaMKK2) pathways [68]. One possible source is Aβ engagement of RAGE, which elevates reactive oxygen species and cytosolic Ca²⁺ to activate the CaMKK2-AMPK axis [69]. While Aβ oligomers have been shown in several cell types to form Ca²⁺-permeable pores that can rapidly increase cytosolic Ca²⁺ [70], this mechanism is unlikely to contribute here, as monomeric Aβ40 is expected to be stable in the cell culture medium over 1 h and no AMPK activation was observed with Aβ42.

### Integrating DMSO-associated Akt activation into interpretation of Aβ effects

DMSO is a widely used solvent for preparing soluble synthetic Aβ [22, 71]. The present study identified DMSO-associated Akt activation in the vehicle − untreated comparison (Fig. 3), which limits interpretation of basal Akt phosphorylation after Aβ exposure and reduces confidence in quantitative interpretation of effect magnitude.

DMSO has been shown in other studies to stimulate Akt phosphorylation via a PI3K-dependent mechanism [36, 72], though the initiating transduction event remains unidentified. One possibility is that DMSO perturbs membrane lipid order and raft organization, potentially activating PI3K/Akt through redistribution of raft-associated kinases such as Src, FAK, and integrins [73–75]. These considerations underscore the need to account for solvent effects and to evaluate alternative Aβ preparation methods, as both the solvent and Aβ appear to exert confounding influences on Akt signaling. Alternative Aβ solubilization approaches that minimize solvent-associated signaling effects while preserving defined aggregation states are needed.

### Experimental scope: pathway discovery and limits of mechanistic inference

This study was designed for hypothesis generation through unbiased profiling of hundreds of proteins across multiple treatment conditions. RPPA enables this breadth but provides semi-quantitative measures with limited dynamic range and precision. In addition, the small sample size (*n* = 3) across seven treatment groups limited statistical power. We applied a conservative analytical strategy appropriate to these platform and design characteristics that emphasized false positive control over sensitivity. Accordingly, the results are interpreted as identifying robust qualitative signaling patterns and candidate pathways for targeted validation rather than providing definitive quantitative characterization of effect size or temporal dynamics. Despite these limitations, the principal Aβ40 insulin-response effects on Akt and AMPK-associated nodes were supported by multiple independent contrasts and remained highly significant after multiple-testing correction, supporting the robustness of the observed qualitative patterns. Our batch design prioritized minimizing technical noise in insulin-response comparisons by running each of these contrasts in one batch, eliminating the potential for cross-batch effects in the principal second-order insulin-response contrasts that support the main findings on Aβ effects on insulin action. This came at the expense of inter-batch variability in non-insulin contrasts; nonetheless, we observed some trends in Aβ-only effects that warrant further investigation.

These observations require validation using orthogonal, higher-resolution methods, as well as mechanistic interrogation through targeted inhibitor studies and functional assays. Beyond validation, several aspects of experimental design merit extension. Aβ aggregation states were inferred from well-characterized preparation protocols, but direct measurement would strengthen the mechanistic interpretation of isoform-specific effects. Single-timepoint measurements (1 h Aβ exposure; 10 min insulin stimulation) provide a defined snapshot of pathway processes but do not capture sequential steps, transient early events, delayed responses, or feedback and recovery dynamics. Single concentration exposure of insulin (100 nM) and Aβ (1 µM)—selected based on empirically determined sensitivity of cell culture models—precludes evaluation of dose-dependent responses that would provide additional mechanistic insight.

### Conclusions: monomeric Aβ40 disruption of BBB endothelial insulin signaling

This exploratory study identified isoform-specific qualitative patterns in acute Aβ effects on BBB endothelial insulin signaling, generating testable mechanistic hypotheses while also revealing the need to optimize Aβ solvent and iBMEC culture media for future investigations. Metabolic insulin signaling (Akt and key downstream branches) emerged as the most substantially affected pathway from unbiased profiling of hundreds of proteins. Testable mechanistic hypotheses include: (1) monomeric Aβ40 disrupts insulin-responsive Akt signaling through interaction with INSR; and (2) attenuation of insulin-stimulated Akt activation contributes to failure of insulin-mediated AMPK suppression, potentially combined with additional AMPK-activating inputs. These findings indicate that Aβ40 acutely disrupts BBB endothelial insulin signaling and provide a mechanistic foundation to guide development of therapeutic approaches for vascular insulin resistance in AD.

## Supplementary Information

Below is the link to the electronic supplementary material.


Supplementary Material 1



Supplementary Material 2



Supplementary Material 3



Supplementary Material 4



Supplementary Material 5


## Data Availability

All data generated or analyzed during this study are included in this published article and its supplementary information files. Processed RPPA expression matrices generated by the MD Anderson Functional Proteomics RPPA Core are included as supplementary files, together with downstream differential expression results for all contrasts.
