# Supplementary material for: Acute amyloid-β exposure disrupts insulin signaling in hCMEC/D3 blood–brain barrier endothelial cells
Source: Fluids Barriers CNS. 2026 Jun 19;23:106. doi: 10.1186/s12987-026-00825-2 (PMC13536614; doi:10.1186/s12987-026-00825-2)
Supplement: Supplementary file 1 [file 12987_2026_825_MOESM4_ESM.docx]

# supplementary material

Supplementary List 1 : Removed probes unrelated to BBB endothelial biology: AR, Aurora.B, B7.H4, CD134, CD20, CD4, CD45, Claudin.7, c.Kit, E.Cadherin, EMA, ER, ER.a_pS118, GATA3, Granzyme.B, HER2, HER2_pY1248, HER3, HER3_pY1289, Heregulin, Lck, MS12, NAPSIN.A, Oct.4, P.Cadherin, PD.1, PR, Rab25, Syk, Tau, TTF1, UGT1A, UBAC1, ZAP.70, PLC.gamma2_pY759, MYH11, PDGFR.b, Notch3.

**Supplementary Figure 1: Silver-stained SDS–PAGE of Aβ preparations.** Aβ samples were prepared as described in Methods and resolved on a 4–15% gradient gel followed by silver staining. Lane assignments: (1) DMSO, (2) F12 medium, (3) vehicle (DMSO + F12, pH 8), (4–6) Aβ40 at 1, 2, and 4 μg, respectively, (7) empty, (8–9) Aβ42 at 1 and 2 μg, respectively, and (10) Aβ40 at 4 μg. Molecular weight markers (kDa) are shown on the right.

Supplementary Table 1: Sample-level quality control metrics and the resulting Limma weights for the hCMEC/D3 contrasts identified as having reduced group coherence and increased within-group variability based on having Mahalanobis distances greater than 2.5 or negative Silhouette widths. The Limma weight applied to each sample is listed in the final column.

| **Dataset** | **Contrast** | **Sample** | **Group** | **Mahalanobis dist.** | **Silhouette width** | **Weight** |
| --- | --- | --- | --- | --- | --- | --- |
| HD3 | ab40ins_vs_ab40 | x7_3a_abeta_40_1hr | ab40 | 0.04695 | 0.19761 | 1.20 |
| HD3 | ab40ins_vs_ab40 | x8_3b_abeta_40_1hr | ab40 | 2.12002 | 0.28934 | 1.05 |
| HD3 | ab40ins_vs_ab40 | x9_3c_abeta_40_1hr | ab40 | 1.73742 | 0.14479 | 1.09 |
| HD3 | ab40ins_vs_ab40 | x10_4a_abeta_40_insulin | ab40_insulin | 2.63089 | -0.07614 | 0.33 |
| HD3 | ab40ins_vs_ab40 | x11_4b_abeta_40_insulin | ab40_insulin | 0.60798 | 0.16011 | 1.68 |
| HD3 | ab40ins_vs_ab40 | x12_4c_abeta_40_insulin | ab40_insulin | 0.85674 | 0.13219 | 1.31 |
| HD3 | ab42ins_vs_ab42 | x13_5a_abeta_42_1hr | ab42 | 0.46365 | 0.16072 | 1.02 |
| HD3 | ab42ins_vs_ab42 | x14_5b_abeta_42_1hr | ab42 | 0.00866 | 0.21246 | 2.09 |
| HD3 | ab42ins_vs_ab42 | x15_5c_abeta_42_1hr | ab42 | 0.36650 | 0.17958 | 1.11 |
| HD3 | ab42ins_vs_ab42 | x16_6a_abeta_42_insulin | ab42_insulin | 2.22765 | -0.06779 | 0.43 |
| HD3 | ab42ins_vs_ab42 | x17_6b_abeta_42_insulin | ab42_insulin | 2.66262 | 0.04448 | 1.15 |
| HD3 | ab42ins_vs_ab42 | x18_6c_abeta_42_insulin | ab42_insulin | 2.27092 | -0.00558 | 0.84 |

Supplementary Figure 2: Decision tree for interpreting modulation of insulin response by Aβ using second-order and level contrasts. Contrasts are I = (insulin − untreated), Iₐ = (insulin with Aβ - Aβ), L = (Aβ+insulin - insulin), and S = Iₐ − I. Each contrast is discretized with the same scoring thresholds used in **Error! Reference source not found.**. Nonzero values require |log₂ fold-change| > 0.138 and FDR < 0.1 (sign from LFC). The tree assigns: no change (S = 0); augmentation (sgn(S) = sgn(L)); reversal (sgn(Iₐ) ≠ sgn(I)); or attenuation when sgn(S) = sgn(I) = sgn(L). Attenuation strength is qualified as robust if |S| = |L| = 100 Cases with L = 0 under the attenuation branch are labeled “possible attenuation or saturation/floor.”

Supplementary Table 2: Literature-based canonical direction of acute insulin response for each measured phosphosite. Mechanisms following insulin stimulation are the cited. Symbols: ↑ increase; ↓ decrease; ↔ no consistent change; ↑/↔ increase or no change; not decrease; ? insufficient or conflicting evidence.

| Probe | Direction | Mechanism following insulin stimulation | Source |
| --- | --- | --- | --- |
| Akt Ser473 | ↑ | PI3K activation leads to mTORC2 phosphorylation of Akt | [1] |
| Akt Thr308 | ↑ | PDK1 phosphorylates Akt at Thr308 | [1] |
| GSK3α/β Ser21/9 | ↑ | Akt phosphorylates and inhibits GSK3 | [1] |
| IGF1R Tyr1135/1136 | ↑↔ | Low-affinity insulin binding can activate IGF1R at high concentration | [2] |
| Gys Ser641 | ↓ | Akt inhibits GSK3, reducing GS Ser641/645 phosphorylation | [3] |
| FoxO3a Ser318/321 | ↑ | Akt phosphorylation inhibits FOXO activity | [1] |
| PDK1 Ser241 | ↔ | PDK1 Ser241 phosphorylation is constitutive and required for activity | [4] |
| S6 Ser235/236 | ↑ | Phosphorylation by S6K downstream of mTORC1 | [1] |
| S6 Ser240/244 | ↑ | Phosphorylation by S6K downstream of mTORC1 | [1] |
| S6K Thr389 | ↑ | Phosphorylation by mTORC1 downstream of Akt-mediated TSC2 inhibition | [1] |
| TSC2 Thr1462 | ↑ | Akt phosphorylation inhibits TSC2 | [1] |
| PRAS40 Thr246 | ↑ | Akt phosphorylates PRAS40 | [1] |
| mTOR Ser2448 | ? | Context-dependent phosphorylation; not a specific mTORC1 activity marker | [1] |
| Rictor Thr1135 | ? | Context-dependent feedback phosphorylation downstream of S6K | [1] |
| HSP27 Ser82 | ↑↔ | MAPK-associated node; context-dependent insulin ERK signaling | [5] |
| SHP2 Tyr542 | ↑↔ | Context-dependent phosphorylation upstream of Ras–ERK signaling | [6] |
| MAPK Thr202/Tyr204 | ↑↔ | ERK activation shows greater dose- and time-dependence than Akt activation | [7,8] |
| MEK1 Ser217/221 | ↑↔ | ERK activation shows greater dose- and time-dependence than Akt activation | [7,8] |
| p90RSK Thr573 | ↑↔ | ERK activation shows greater dose- and time-dependence than Akt activation | [7,8] |
| C-Raf Ser338 | ↑↔ | ERK activation shows greater dose- and time-dependence than Akt activation | [7,8] |
| Shc Tyr317 | ↑↔ | Shc Tyr317 links insulin signaling to Grb2–MAPK activation | [9] |
| YB1 Ser102 | ↑↔ | YB1 Ser102 phosphorylation downstream of RSK signaling | [10] |
| AMPKα Thr172 | ↓ | Akt suppresses AMPK activation | [1] |
| ULK1 Ser757 | ↑ | mTORC1 phosphorylates ULK1 Ser757, opposing AMPK activation | [11] |
| ACC Ser79 | ↓ | AMPK phosphorylates ACC at Ser79 | [12] |

1. Manning BD, Toker A. AKT/PKB Signaling: Navigating the Network. Cell. Elsevier; 2017;169:381–405. https://doi.org/10.1016/j.cell.2017.04.001

2. Steele-Perkins G, Turner J, Edman JC, Hari J, Pierce SB, Stover C, et al. Expression and characterization of a functional human insulin-like growth factor I receptor. Journal of Biological Chemistry. Elsevier; 1988;263:11486–92. https://doi.org/10.1016/S0021-9258(18)37983-3

3. Bouskila M, Hirshman MF, Jensen J, Goodyear LJ, Sakamoto K. Insulin promotes glycogen synthesis in the absence of GSK3 phosphorylation in skeletal muscle. Am J Physiol Endocrinol Metab. 2008;294:E28-35. https://doi.org/10.1152/ajpendo.00481.2007

4. Komander D, Kular G, Deak M, Alessi DR, Aalten DMF van. Role of T-loop Phosphorylation in PDK1 Activation, Stability, and Substrate Binding *. Journal of Biological Chemistry. Elsevier; 2005;280:18797–802. https://doi.org/10.1074/jbc.M500977200

5. Kostenko S, Moens U. Heat shock protein 27 phosphorylation: kinases, phosphatases, functions and pathology. Cell Mol Life Sci. 2009;66:3289–307. https://doi.org/10.1007/s00018-009-0086-3

6. Araki T, Nawa H, Neel BG. Tyrosyl phosphorylation of Shp2 is required for normal ERK activation in response to some, but not all, growth factors. J Biol Chem. 2003;278:41677–84. https://doi.org/10.1074/jbc.M306461200

7. Wang Z, Tang X, Swaminathan SK, Kandimalla KK, Kalari KR. Mapping the dynamics of insulin-responsive pathways in the blood–brain barrier endothelium using time-series transcriptomics data. npj Syst Biol Appl. Nature Publishing Group; 2022;8:29. https://doi.org/10.1038/s41540-022-00235-8

8. Kubota H, Noguchi R, Toyoshima Y, Ozaki Y, Uda S, Watanabe K, et al. Temporal Coding of Insulin Action through Multiplexing of the AKT Pathway. Molecular Cell. 2012;46:820–32. https://doi.org/10.1016/j.molcel.2012.04.018

9. Ishihara H, Sasaoka T, Wada T, Ishiki M, Haruta T, Usui I, et al. Relative involvement of Shc tyrosine 239/240 and tyrosine 317 on insulin induced mitogenic signaling in rat1 fibroblasts expressing insulin receptors. Biochem Biophys Res Commun. 1998;252:139–44. https://doi.org/10.1006/bbrc.1998.9621

10. Stratford AL, Fry CJ, Desilets C, Davies AH, Cho YY, Li Y, et al. Y-box binding protein-1 serine 102 is a downstream target of p90 ribosomal S6 kinase in basal-like breast cancer cells. Breast Cancer Res. 2008;10:R99. https://doi.org/10.1186/bcr2202

11. Kim J, Kundu M, Viollet B, Guan K-L. AMPK and mTOR regulate autophagy through direct phosphorylation of Ulk1. Nat Cell Biol. 2011;13:132–41. https://doi.org/10.1038/ncb2152

12. Hardie DG, Ross FA, Hawley SA. AMPK: a nutrient and energy sensor that maintains energy homeostasis. Nat Rev Mol Cell Biol. 2012;13:251–62. https://doi.org/10.1038/nrm3311

Supplementary Figure 3: **Western blot analysis of Akt Ser473 phosphorylation following Aβ40 exposure and insulin stimulation.** Western blots for pAkt Ser473 and total Akt are shown. Quantification of pAkt Ser473 normalized to total Akt is presented as fold change relative to the corresponding no-insulin condition within each treatment arm. Data are shown as mean ± SD (n = 3). Significance is indicated as *p < 0.05, **p < 0.01, ***p < 0.001, ****p < 0.0001. Statistical analysis was performed using two-way ANOVA with Aβ40 and insulin as factors, followed by three pairwise comparisons with Holm–Sidak correction. Adjusted p-values were: control + insulin vs control, p = 1.91×10⁻⁶; Aβ40 + insulin vs Aβ40, p = 0.0189; control + insulin vs Aβ40 + insulin, p = 7.61×10⁻⁶. Mean fold changes (log2 insulin response) were 1.47 (vehicle) and 0.45 (Aβ40), corresponding to a 1.03-unit reduction in insulin responsiveness.

Supplementary Figure 4: Systems Biology Graphical Notation (SBGN) Process Description graph of metabolic insulin signaling and related processes that includes measured RPPA probes in white and nodes without corresponding probes in grey.
